# Overexpression of miR-148a-3p inhibits extracellular matrix degradation and alleviates IL-1β-induced intervertebral disc degeneration

**DOI:** 10.22038/IJBMS.2022.64645.14228

**Published:** 2023-02

**Authors:** Hehuan Lai, Jialin Fan, Yejin Zhang, Bin Pan, Wenzheng Pan, Jiawei Fang, Kainan Ni, Zhenzhong Chen, Shijie Liu, Chao Lou, Dengwei He

**Affiliations:** 1 Department of Orthopaedic Surgery, The Fifth Affiliated Hospital of Wenzhou Medical University, Affiliated Lishui Hospital of Zhejiang University,; 2 Lishui Municipal Central Hospital, 289 Kuocang Road, Lishui 323000, Zhejiang Province, China; 3 Lishui Second People’s Hospital, Beihuan Road 69, Liandu District, Lishui323000, Zhejiang Province, China; # These authors contributed equally to this work

**Keywords:** Extracellular matrix, Intervertebral disc - microRNAs, miR-148a-3p, Nucleus pulposus

## Abstract

**Objective(s):**

Recently, studies on microRNAs (miRNAs) and their targets and related genes have provided new therapeutic opportunities for controlling intervertebral disc degeneration (IDD). We aimed to investigate the effects of miR-148a-3p overexpression on IDD progression.

**Materials and Methods:**

This study used microRNA microarrays to analyze key regulators of IDD. Q-PCR was used to verify the IL-1β-induced down-regulation of miR-148a-3p expression both in nucleus pulposus (NP) tissues of IDD patients and in degenerated NP cells (NPCs) of rats. Rat NPC micromass cultures and *ex vivo* intervertebral disc (IVD) culture models were established, and histological staining was performed to verify the effect of miR-148a-3p on the general morphology and proteoglycan and collagen contents of IVDs. In addition, q-PCR and western blotting analyses were performed to examine the expression of ECM molecules and matrix-degrading enzymes. A luciferase reporter assay was used to confirm the target genes of miR-148a-3p.

**Results:**

Our data revealed that miR-148a-3p was down-regulated in IDD. Overexpression of miR-148a-3p had no effect on ACAN or COL2A1 gene expression but decreased MMP3, MMP13, and ADAMTS5 gene expression. The matrix deposited by miR-148a-3p-overexpressing rat NPCs contained high levels of proteoglycans and collagen. The* ex vivo* experiments verified that agomiR-148a-3p alleviated the NPC matrix degradation induced by IL-1β. A luciferase reporter assay confirmed that miR-148a-3p directly targeted ADAMTS5 and MMP13.

**Conclusion:**

We proved that miR-148a-3p can attenuate ECM loss and protect NP function by inhibiting matrix-degrading enzymes.

## Introduction

Low back pain (LBP) is a leading cause of disability worldwide and causes substantial clinical and socioeconomic burdens (1). Although numerous potential causes have been recognized, intervertebral disc degeneration (IDD) is the most common cause of LBP (2, 3). Degeneration of the nucleus pulposus (NP), which is the central gel-like part of an intervertebral disc (IVD), is a major contributor to disc degeneration (4, 5). NP cells (NPCs) are the principal cell type in the NP and are critical for secreting a complex extracellular matrix (ECM) that is mainly composed of collagens and proteoglycans. During IDD, NPCs undergo a phenotypic transition (transitioning from an effector NPC phenotype to a fibro-NPC phenotype) (6), leading to increased production of catabolic proteases and decreased synthesis of ECM components. The increase in catabolic proteases, including matrix metalloproteinases (MMPs), disintegrins, and metalloproteinases with thrombospondin motifs (ADAMTSs), along with proinflammatory cytokines disrupts ECM homeostasis, and ECM undergoes profound metabolic changes (7). In addition, ECM degradation products may further promote the inflammatory response that is related to IDD. The current conservative and surgical therapeutic options have limited treatment efficacy and usually fail to reverse pathological alterations or stop the degenerative progress (8).

MicroRNAs (miRNAs) are small (20–22 nucleotides long), tissue-specific noncoding RNA (ncRNA) molecules that play central roles in cell differentiation, proliferation, and survival by binding to complementary target mRNAs, resulting in mRNA translational inhibition or degradation (9). Increasing evidence suggests a role for miRNAs in degenerative disc disease (10). Multiple miRNAs are differentially expressed in human degenerated intervertebral disc tissues and have been verified to be associated with pathological processes, including NPC apoptosis, ECM degradation, and inflammatory responses (11-14). Since the discovery of their clinical therapeutic potential, certain miRNAs have been translated from bench to bedside, and some have successfully passed phase I trials (15). Therapeutic approaches that manipulate miRNAs provide new insights into and evidence to support new IDD treatments.

MiR-148a was found to be down-regulated in degenerated nucleus pulposus tissues from IVDD patients (16). In addition, another study showed that the expression of miR-148a was down-regulated in peripheral blood mononuclear cells from IVDD patients, and miR-148a may inhibit the release of proinflammatory cytokines from intervertebral disc cells and contribute to IVDD progression (17). It can be seen that miR-148a plays a certain role in regulating intervertebral disc degeneration, but the specific downstream mechanism is not completely clear. Whether miR-148a-3p plays a role in nucleus pulposus cells in IDD and whether miR-148a-3p has other target genes associated with IDD are still unknown.

In this study, we found that the miR-148a-3p expression level was significantly decreased in IDD patients and that miR-148a-3p may play a critical role in inhibiting IDD by repressing *ADAMTs5* and *MMP13* expression.

## Materials and Methods

***Microarray data information and processing of differentially expressed miRNAs (DEMs)***

The National Center for Biotechnology Information-Gene Expression Omnibus (NCBI-GEO) is a free public database of microarray profiles, and we obtained the gene expression profile from the GSE45856 dataset (based on the GPL11434 platform). The microarray data in the GSE45856 dataset include data from 3 degenerated NP tissue samples and 3 normal NP tissue samples. DEMs between the degenerated NP specimens and normal NP specimens were identified via GEO2R online tools on the basis of a |log2 fold change (FC)| > 2 and an adjusted *P*-value<0.05. The differentially expressed miRNAs between degenerated NP tissues and normal NP tissues were analyzed by using R software (3.6.3) with the “limma” package, and the “pheatmap” package was used to generate heatmaps. Based on a volcano plot and filtering based on fold change, we identified DEMs in the IDD samples.

***Human samples***

This study was approved by the Ethics Committee of the Lishui Central Hospital (2020-414). Twelve clinical samples were collected for this project. All NP tissue samples were obtained from the Lishui Central Hospital (Zhejiang, China). Informed consent was obtained from all subjects. Among the 12 patients, 6 patients showed significant disc degeneration with Pfirrmann’s criterion grade >III, and these patients underwent surgery for discectomy and fusion and were enrolled in the IDD group. Additionally, 6 spinal trauma patients without signs of disc degeneration underwent vertebral body and disc excision and fusion and were enrolled in the control group. Human IVD tissue and control tissue were obtained from the cervical portion of the spine of patients undergoing surgery.

***NP cell separation and culture***

The human disc tissue specimens were first washed twice with phosphate-buffered saline (PBS), and the NP was separated from the AF using a stereotaxic microscope and cut into small fragments (2–3 mm^3^). The fragments were digested with 0.2% type II collagenase (Sigma, United States) for 12 hr and filtered through a cell strainer, and the isolated cells were rinsed twice with PBS. The cells were then cultured with complete culture medium (DMEM/F12, Gibco, United States) supplemented with 10% fetal bovine serum (FBS, Gibco, United States) and antibiotics (100 mg/ml streptomycin and 100 U/ml penicillin) in 5% CO2 and at 37 **°**C. The medium was changed every 2–3 days.

***Animals***

Twelve-week-old C57BL/6J mice were purchased from Shanghai Silaike Experimental Animals Center (Shanghai, China). All of the animals were housed under standard environmental conditions (23 ± 2 °C, 12-hour light/dark cycle) with free access to food, and they were allowed to move freely. The animal experiments conformed to the Principles of Laboratory Animal Care (National Society for Medical Research), and extensive efforts were made to minimize the suffering of the animals included in the study.

***Micromass culture***

Rat NPCs were kindly prepared and donated by Prof. Fengdong Zhao (Zhejiang University) (18). The cells were seeded in a 24-well culture plate at a density of 5×10^6^ cells/ml in a final volume of 10 μl of DMEM. After the cells were allowed to adhere to the culture dishes for 4 hr at 37 °C in a humidified atmosphere containing 5% CO_2_, 0.5 ml of DMEM/F12 supplemented with 10% fetal bovine serum (FBS), 25 U penicillin/ml, 25 μg streptomycin/ml, and 0.5 mM glutamine was added. The following day, the cells were transfected with a miR-148a mimic or negative control for 24 hr and then stimulated with or without IL-1β (10 ng/ml) for 48 hr. Then, the micromass cultures were washed once with PBS and fixed for one hour at room temperature with 0.5 ml of 4% paraformaldehyde. The cell masses were subsequently washed three times with deionized water and stained with safranin O, tolonium chloride, and Alcian blue. The absorbance of the cell mass was measured at 554 nm (safranin O), 629 nm (tolonium chloride), and 600 nm (Alcian blue).

***Ex vivo*** ***culture***

Lumbar spine segments were obtained from C57BL/6J mice and dissected under sterile conditions within 1 hr of sacrifice for *ex vivo* organ culture in DMEM/F-12 medium (19, 20). Lipofectamine 3000 Transfection Reagent (Invitrogen) was used to transfect the cultured spine segments with agomiR-148a-3p or controls at 200 nM. After 24 hr, the spine segments were cultured in the presence or absence of IL-1β (10 ng/ml) for 48 hr. The harvested spine segments were fixed in 4% paraformaldehyde and decalcified in EDTA solution, which was changed every 4 days. 

***Histological staining***

Decalcified spine segments were embedded in paraffin. Serial disc sections of exactly 5 μm thickness were generated to prepare slides. Hematoxylin and eosin (H&E) staining and safranin O/fast green staining were performed to assess the general morphology and visualize the proteoglycans and type II collagens in the discs. Briefly, for safranin O/fast green staining, the sections were deparaffinized, rehydrated, stained with 1% fast green solution for 10 min at room temperature, and washed with water to remove excess dye until the cartilage was colorless. Then, the sections were counterstained with safranin O solution for 30 sec, followed by differentiation in 1% HCl-ethanol. For H&E staining, the sections were stained with hematoxylin for 2 min and rinsed with running water for 10 sec, followed by color separation with 1% HCl-ethanol. Then, the sections were rinsed under running water, stained with eosin for 1 min, and washed with distilled water for 10 sec. Finally, the sections were dehydrated twice with 95% and 100% ethanol for 1 min, permeabilized with xylene, mounted with neutral gum, and air-dried. The safranin O staining of the ECM of the NPCs was quantified based on the integrated optical density (IOD) using ImageJ software.

***RNA isolation and q-PCR***

Total RNA was extracted from cultured rat NPCs or human NPCs using an RNA-Quick purification kit (Yishan Biotech, Shanghai, China) according to the manufacturer’s instructions. The RNA was reverse transcribed into cDNA using a HiFiScript cDNA synthesis kit (CWBio, Beijing, China) and a miRNA first-strand cDNA synthesis kit (stem–loop) (Vazyme, Nanjing, China). The obtained cDNA was then amplified on an ABI 7500 System (Applied Biosystems, Foster City, CA, USA) with qPCR SYBR Green Master Mix (Yishan Biotech, Shanghai, China) and miRNA Universal SYBR qPCR Master Mix (Vazyme, Nanjing, China), and β-actin and U6 RNA (for the miRNAs) served as the reference genes. To analyze the results, relative expression was calculated using the 2^-ΔΔCT^ method. The primers used for PCR are shown in Tables 1 and 2.

***Western blotting***

Cells were lysed with RIPA lysis buffer (Solarbio, Beijing, China) and centrifuged at 14,000 rpm and 4 °C to obtain the supernatants. The protein concentration was quantified using a BCA protein assay kit (Beyotime, Shanghai, China). The proteins were separated by electrophoresis and then transferred to PVDF membranes (Millipore, Boston, MA, USA). Then, the membranes were blocked with 5% skim milk for 2 hr at room temperature, washed three times with TBST, and incubated with primary antibodies overnight at 4 °C. Then, the cells were washed with TBST and incubated with horseradish peroxidase (HRP)-labeled goat anti-rabbit/anti-mouse IgG secondary antibody (Danvers, MA, USA) for 1 hr at room temperature. The protein bands were visualized using an iBright FL1000 system (Thermo Fisher Scientific, Waltham, MA, USA) and quantitatively analyzed with ImageJ software (National Institutes of Health, Bethesda, MD, USA). Primary antibodies against MMP3 (ab52915) and COL2A1 (ab188570) were obtained from Abcam (Cambridge, UK); an antibody against MMP13 (ET1702-14) was obtained from Huabio (Hangzhou, China); antibodies against ACAN (A8536), ADAMTS4 (A2525), and ADAMTS5 (A2836) were obtained from ABclonal (Wuhan, China); and antibodies against β-actin (#3700T) were obtained from Cell Signaling Technology (Danvers, MA, USA).

***Dual luciferase assay***

The pMIR vector (Ambion, USA) was used to express luciferase and was used as the template vector for 3’-UTR reporter assays. Wild-type and mutant sequences of the 3′-UTRs of the *ADAMTs5* and *MMP13* mRNAs were cloned and inserted into the 3′-UTR of the pMIR vector. HEK293 cells were seeded in a 96-well plate and then transfected with the indicated microRNA mimic molecules, 100 ng reporter plasmid pMIR-Report construct or control vector, and 10 ng internal control pRL-TK Renilla luciferase plasmid with Lipofectamine 2000 following the manufacturer’s instructions. After 48 hr, the Dual Luciferase Assay System (Promega) was used to measure reporter activity.

***Statistical analysis***

All the research data are expressed as the means ± SDs based on at least three experiments. One-way ANOVA and Student’s t-test were performed for data analysis using GraphPad Prism software (GraphPad Software, La Jolla, CA, USA). A value of *P*<0.05 indicated a significant difference.

## Results

***miR-148a-3p is down-regulated in degenerated NPCs***

**Figure 1:**
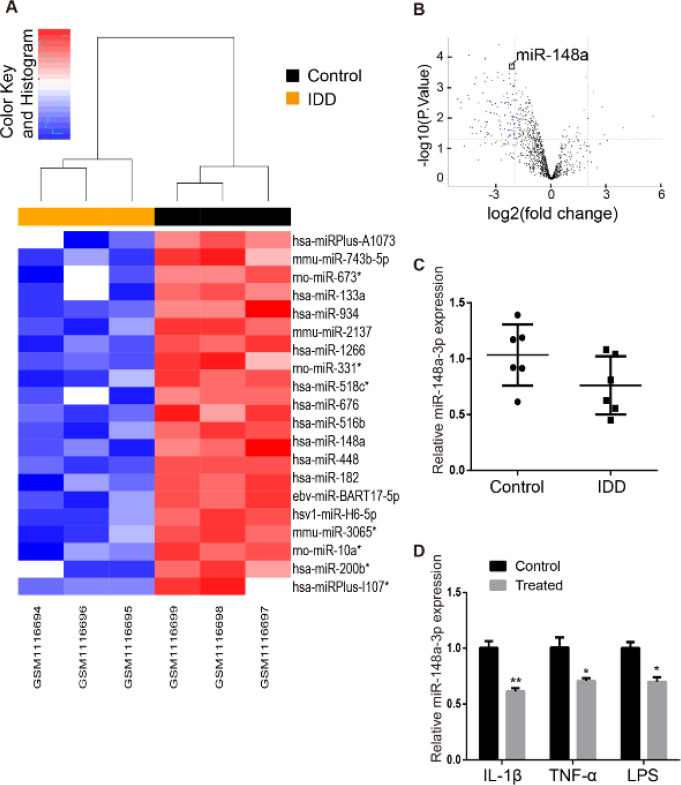
miR-148a-3p is down-regulated in IDD patients

In the current study, we first obtained the gene expression profile from the GSE45856 dataset in the GEO database. The microdata of the GSE45856 dataset was obtained on the basis of GPL11434 platforms (miRCURY LNA microRNA Array), which included human NP tissue samples (3 IDD patient samples vs 3 normal control samples). The results of an unsupervised clustering analysis of the miRNAs are shown in a volcano plot and heatmap (Figure 1A, B). The miR-148a-3p levels were significantly imbalanced (adjusted *P*<0.05 and |log2FC| > 2). We used qRT–PCR experiments to analyze the expression patterns of miR-148a-3p in NP tissue samples obtained from 6 IDD patients and normal tissue samples from 6 patients with idiopathic scoliosis. The analysis results showed that the miR-148a-3p level was reduced in the NP tissues of the IDD patients compared with the NP tissues of normal subjects (Figure 1C). An *in vitro* study showed that miR-148a-3p is expressed at a low level in rat NPCs treated with 10 ng/ml IL-1β, 10 µg/ml lipopolysaccharide (LPS), or 25 ng/ml TNF-α for 48 hr (Figure 1D) (21).

***The effect of miR-148a-3p overexpression on the NPC phenotype***

**Figure 2:**
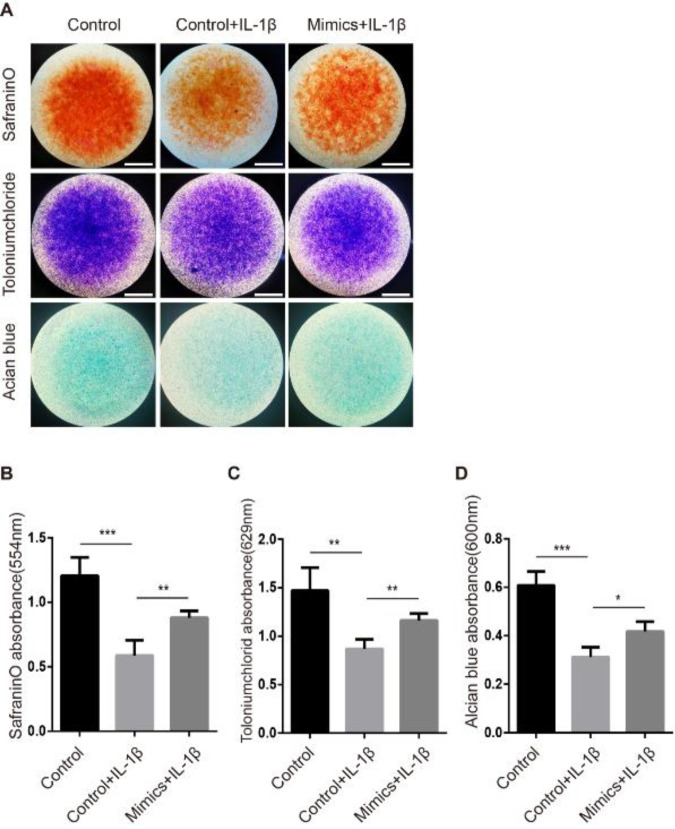
miR-148a-3p mimics attenuate IL-1β-induced extracellular matrix (ECM) loss in nucleus pulposus cells (NPCs)

To further elucidate the effect of miR-148a-3p on rat NPCs, a miR-148a-3p mimic was transfected into NPC micromass cultures and incubated for 24 hr, and then, these cells were treated with or without IL-1β (10 ng/ml) for 48 hr. We measured matrix production by safranin O, tolonium chloride, and Alcian blue staining. As expected, the miR-148a-3p mimic alleviated the ECM loss that was caused by IL-1β, as indicated by the increase in safranin O, tolonium chloride, and Alcian blue staining (Figure 2).

***miR-148a-3p overexpression alleviates NPC ECM degradation***

**Figure 3:**
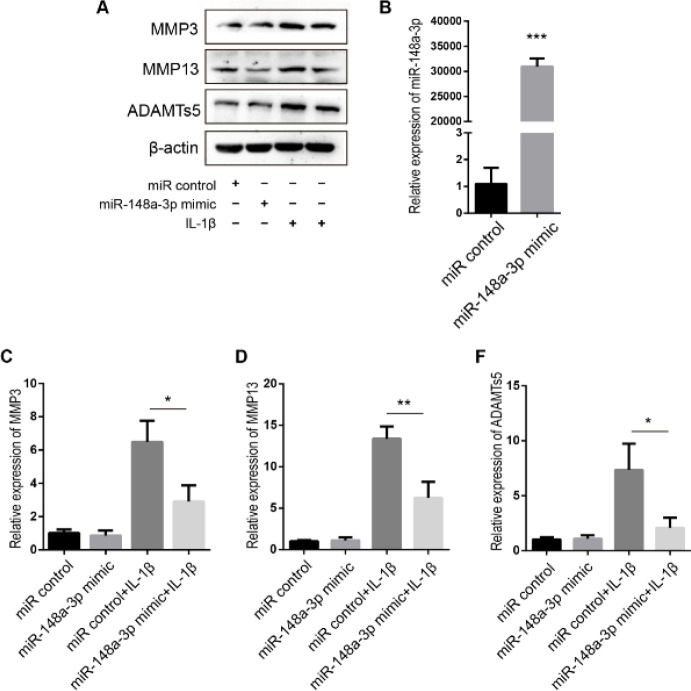
miR-148a-3p mimics inhibit the levels of matrix-degrading enzymes

We further studied the effects of miR-148a-3p overexpression on ECM components and matrix-degrading enzymes. The transfection efficiency of the miR-148a-3p mimics was evaluated, and the results are shown in Figure 3B. IL-1β stimulation was used to induce NPC degeneration (22). The results demonstrated that compared with those in the miR-148a-3p mimic control-transfected NPCs, the levels of MMP3, MMP13, and ADAMTs5 were decreased in the NPCs transfected with miR-148a-3p mimics (Figure 3A, C, D, F). Overall, the data show that overexpression of miR-148a-3p inhibits the degradation of the NPC matrix that is caused by IL-1β.

***Protective effect of miR-148a-3p on mouse IVDs***

**Figure 4:**
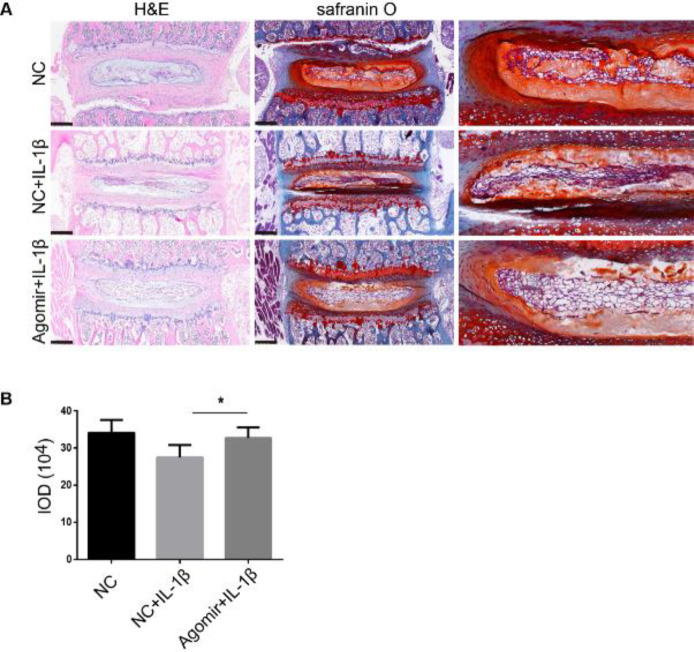
Agomir-148a-3p attenuates IL-1β-induced extracellular matrix (ECM) loss in ex vivo cultured murine intervertebral discs (IVDs)

To verify the protective effect of miR-148a-3p on disc degeneration, lumbar spine segments obtained from C57BL/6J mice were cultured *ex vivo* and transfected with 200 nM agomir-148a-3p or agomir control. IL-1β stimulation was used to induce IDD. H&E and safranin O staining were performed to assess the general morphology and visualize the proteoglycans and type II collagens in the discs. As shown in Figure 4, more intense and larger areas of red staining were observed in the NP tissues in the agomir-148a-3p group than in those in the control group. Safranin O staining indicated that agomir-148a alleviated the ECM loss that was stimulated by IL-1β in mouse intervertebral discs.

Luciferase reporter assay confirmed that miR-148a-3p directly targeted *ADAMTS5* and *MMP13.*

**Figure 5:**
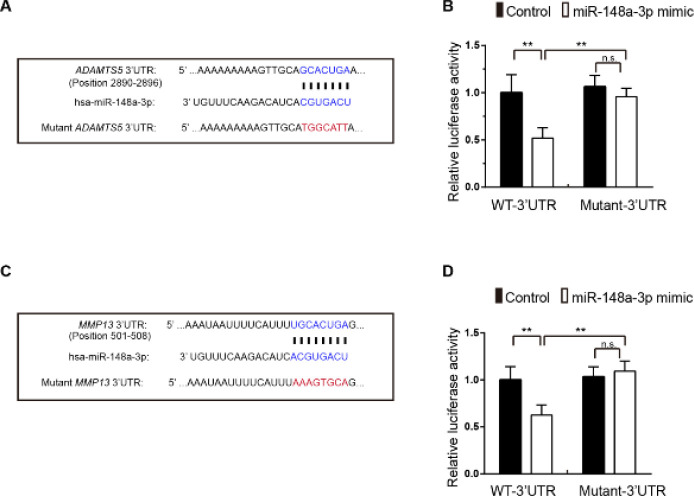
ADAMTS5 and MMP13 are direct targets of miR-148a-3p

According to the effects of miR-148a-3p overexpression on the expression of ECM components and matrix-degrading enzymes at the mRNA and protein levels, we analyzed the sequences of miR-148a-3p and *ADAMTS5* and *MMP13* mRNAs using the TargetScan and miRDB online microRNA target prediction tool. The results showed that miR-148a-3p had sequence complementarity to and could bind to *ADAMTS5* and *MMP13* mRNA sequences (Figure 5A, C). Furthermore, we performed a 3’ UTR luciferase reporter assay and found that the miR-148a-3p mimic decreased the luciferase activity of *ADAMTS5* and *MMP13* reporter 3’ UTRs (Figure 5B, C). Mutation of the miR-148a-3p binding region in the 3’ UTR of *ADAMTS5* or *MMP13* mRNA abolished the regulatory effect (Figure 5B, C). These results confirm that *ADAMTS5* and *MMP13* are directly targeted by miR-148a-3p.

**Table 1:** Primers used for q-PCR of rat

mRNA	Forward primer	Reverse primer
β-actin	TACAACTCCTTGCAGCTCC	ATCTTCATGAGGTAGTCAGTC
MMP 3	TCTTCCTCTGAAACTTGGCG	AGTGCTTCTGAATGTCCTTCG
MMP 13	GTGACTCTTGCGGGAATCCT	CAGGCACTCCACATCTTGGT
ADAMTS 4	CTTCGCTGAGTAGATTCGTGG	AGTTGACAGGGTTTCGGATG
ADAMTS 5	GGGTTATACTGACGTTGTGAGG	TCTAGTCTGGTCTTTGGCTTTG
COl2A1	AGTCCAGTGTGTAGCGTGTG	ACCCCTCTCTCCCTTGTCAC
ACAN	GCGATGCCACCTTGGAAATC	AGTCCAGTGTGTAGCGTGTG

**Table 2:** Primer sequences of miRNA used in this study for q-PCR of human and rat

miRNA	Primer sequence (5′- 3′)
U6 (Rat)	RT: CGCTTCACGAATTTGCGTGTCAT Forward: GCTTCGGCAGCACATATACTAAAAT Reverse: CGCTTCACGAATTTGCGTGTCAT
U6 (Human)	RT: AACGCTTCACGAATTTGCGT Forward: CTCGCTTCGGCAGCACA Reverse: AACGCTTCACGAATTTGCGT
miR-148a-3p (Rat)	RT:GTCGTATCCAGTGCAGGGTCCGAGGTATTCGCACTGGATACGACCAAAGT Forward: GCGCGTCAGTGCACTACAGA Reverse: AGTGCAGGGTCCGAGGTATT
miR-148a-3p (Human)	RT:GTCGTATCCAGTGCAGGGTCCGAGGTATTCGCACTGGATACGACGAGACA Forward: GATAGAAGTCAGTGCACTACAGAACTT Reverse: AGTGCAGGGTCCGAGGTATT

(A) Heatmap depicting 21 differentially expressed miRNAs |fold change (FC)|>2, adjusted P-value<0.05. (B) Volcano plot illustrating the biological and statistical significance of differential miRNA expression levels between IDD patients and controls. The negative log10-adjusted *P*-values (y-axis) are plotted against the log2-fold changes in expression (x-axis). Blue dots indicate up-regulated microRNAs (miRNAs, right side), and yellow dots indicate down-regulated miRNAs (left side). miR-148a-3p is indicated. (C) miR-148a-3p expression in nucleus pulposus (NP) tissue samples from IDD patients (n=6) and normal tissue samples from idiopathic scoliosis patients (n = 6) were measured by qRT–PCR. (D) Rat NP cells were treated with IL-1β (10 ng/ml), TNF-α (20 ng/ml), or lipopolysaccharide (LPS) (0.1 µg/ml) for 24 hr, and qPCR was used to measure the expression of miR-148a-3p. *P<0.05, **P<0.01 vs the control. The data show the normalized means ± SDs

(A) Rat NPCs were processed for micromass culture in medium, transfected with a miR-148a mimic control or mimic, incubated for 24 hr, and then stimulated with or without IL-1β (10 ng/ml) for 48 hr. The cell masses were stained with safranin O, tolonium chloride, and Alcian blue after 3 days in culture. Magnification, 40× (Leica dfc7000t). (B) Safranin O staining for negatively charged proteoglycans and type II collagen. The absorbance was measured at 554 nm. (C) Tolonium chloride staining for proteoglycan production. The absorbance was measured at 629 nm. (D) Alcian blue staining for proteoglycan production. The absorbance was measured at 600 nm. Scale bar, 400 µm. *P<0.05, **P<0.01, ***P<0.001 vs the control. The data show the normalized means ± SDs

(A) Western blot bands showing the expression levels of the MMP3, MMP13, and ADAMTS5 proteins in rat nucleus pulposus cells (NPCs) transfected with a miR-148a-3p mimics/mimic control treated with or without 10 ng/ml IL-1β. (B) The transfection efficiency of the miR-148a-3p mimic was confirmed by q-PCR. (C) q-PCR analysis of the mRNA transcription levels of MMP3, MMP13, and ADAMTS5. *P<0.05, **P<0.01, ***P<0.001 vs control. The data show the normalized means ± SDs

(A) Hematoxylin and eosin (H&E) and safranin O staining were performed to assess the general morphology and proteoglycan loss of discs (n=3). The images shown at high magnification are representative of the inset images, which were captured at low magnification. Scale bar, 200 µm. (B) The red-stained collagen II- and proteoglycan-rich matrix in the nucleus pulposus (NP) was quantified by integrated optical density (IOD). *P<0.05 vs the control. The data show the normalized means ± SDs

(A) miR-148a-3p binding region in the 3’ untranslated region of human ADAMTS5. (B) Luciferase activities measured in HEK293 cells cotransfected with luciferase reporter plasmids carrying the WT or mutant 3’ UTR of ADAMTS5 together with miR-148a-3p mimic or the corresponding control (n=3). (C) miR-148a-3p binding region in the 3’ untranslated region of human MMP13. (D) Luciferase activities measured in HEK293 cells cotransfected with luciferase reporter plasmids carrying the WT or mutant 3’ UTR of MMP13 together with miR-148a-3p mimic or the corresponding control (n=3). **P<0.01 vs the control; n.s. not statistically significant by two-way ANOVA. The data show the normalized means ± SDs

## Discussion

In the present study, we found that dysregulated miR-148a-3p expression plays a critical role in IDD development through its interactions with* ADAMTs5* and *MMP13*. In accordance with miRNA expression profiling via microarray analysis, we found that the expression levels of miR-148a-3p were markedly decreased in degenerated human discs. To verify these results, we collected NP specimens from IDD and idiopathic scoliosis patients and evaluated miR-148a-3p expression by qRT–PCR. Further *in vitro* studies showed that the expression of miR-148a-3p was decreased in rat NPCs stimulated with different proinflammatory cytokines (IL-1β and TNF-α) and LPS. Subsequently, the results of the NPC and micromass culture analyses revealed that miR-148a-3p overexpression decreased MMP3, MMP13, and ADAMTS5 gene and protein expression and ameliorated the ECM loss that was induced by IL-1β. The *ex vivo* culture of murine spine fragments provided further evidence to strengthen the veracity of these results. A luciferase reporter assay confirmed that miR-148a-3p directly targeted *ADAMTS5* and *MMP13*.

Currently, the field of ncRNAs is attracting great attention. MiRNAs may be used to develop promising strategies for the prevention and treatment of IDD (23-26). Here, we describe a miRNA, miR-148a-3p, that was significantly down-regulated in IDD. Previous bioinformatics analysis studies have shown that miR-148a-3p is dysregulated in degenerative NP tissues from IDD patients; however, to the best of our knowledge, only one study has explored the link between miR-148a-3p and IDD, and these authors verified that the levels of miR-148a-3p were decreased in blood samples from IDD patients. A study by Li and colleagues suggested that miR-148a exerts an anti-inflammatory effect and contributes to IDD progression (17). Our findings complement this previous study, and we verified the protective effect of miR-148a-3p on IVDs, especially at the phenotypic level. Using rat NPC micromass culture and *ex vivo* mouse spine culture, we also verified the protective effects of miR-148a-3p at the organizational level. We performed safranin O staining to detect negatively charged proteoglycans and type II collagen, and we performed tolonium chloride and Alcian blue staining to detect proteoglycan production. Notably, according to the staining results, ECM destruction that was induced by IL-1β was attenuated by the miR-148a-3p mimic/agomiR-148a-3p, clearly suggesting that miR-148a-3p is an essential regulator of disc degeneration under pathological conditions.

Moreover, in addition to its role in IDD, the dysregulation of miR-148a-3p in patients with other musculoskeletal diseases, such as osteoporosis and osteoarthritis, has also been reported (27-31). The major new finding presented in this study shows that miR-148a-3p may have anticatabolic effects on NP. The anti-catabolic role was revealed by the result showing that transfection of miR-148a-3p mimics suppressed the IL-1β-mediated induction of ECM-degrading enzymes, including MMP-3, MMP-13, and ADAMTS-5, in rat NPCs. However, we did not observe an effect of miR-148a-3p overexpression on promoting COL2A1 expression in rat NPCs. Since our studies with both rat and murine discs (as biological sources for cells and spinal *ex vivo* cultures) yielded converging results, our findings, in conjunction with other evidence, suggest that miR-148a-3p is an inhibitor that suppresses the progression of IDD.

This study has several known limitations that must be taken into account. First, the findings reported here relate only to *in vitro* and *ex vivo* culture models, which cannot adequately represent the complex variety of factors that may affect IVD homeostasis *in vivo*. In addition, the majority of our experiments were focused on NP cells and may not capture the effects of miR-148a-3p on the entire IVD, which includes the annulus fibrosus and adjacent endplates. Finally, this study showed that miR-148a-3p could directly target and repress *ADAMTS5* and *MMP13* expression in nucleus pulposus cells. However, whether miR-148a-3p exerts a possible effect on other upstream mediators or signaling pathways that mediate the protective effects involved in IDD is also an interesting question that needs to be studied in the future. Does miR-148a-3p affect the transition of NPCs from an effector NPC phenotype to a fibro-NPC phenotype and shift the metabolic process-related genes’ expression[6]? It’s an interesting subject.

## Conclusion

In summary, considering our findings, we hypothesized that miR-148a-3p can attenuate the ECM degradation induced by IL-1β and protect NP function by regulating matrix-degrading enzymes. MiR-148a-3p can directly target and repress ADAMTS5 and MMP13 expression in nucleus pulposus cells. Enhancing miR-148a-3p expression in degenerative discs may have potential therapeutic benefits. These results could improve the understanding of IDD and provide a possible diagnostic marker and therapeutic target for IDD.

## Authors’ Contributions

HL and DH designed the experiments. HL, JF, LZ, YZ, ZC, SL, BP, JF, KN, and CL performed the experiments. JF analyzed data. HL and DH wrote the paper. All the authors read the approved manuscript.

## Conflicts of Interest

The author(s) declare no potential conflicts of interest with respect to the research, authorship, and/or publication of this article.
